# Thiamine Deficiency and Brain Injury: Neuroanatomical Changes in the Wernicke-Korsakoff Syndrome

**DOI:** 10.7759/cureus.92643

**Published:** 2025-09-18

**Authors:** Munirah A Batarfi

**Affiliations:** 1 College of Medicine, Department of Anatomy, King Saud University, Riyadh, SAU

**Keywords:** alcohol-related brain damage, brain imaging, cognitive impairment, korsakoff’s syndrome, memory disorders, neuroanatomy, neuropathology, thiamine deficiency, wernicke’s encephalopathy

## Abstract

Thiamine deficiency triggers an acute neurological emergency, Wernicke’s encephalopathy, which, if left untreated, can progress to the chronic amnestic Korsakoff syndrome. While chronic alcohol misuse remains the classic risk factor, thiamine deficiency is increasingly documented in malnutrition, hyperemesis gravidarum, post-bariatric surgery, chronic gastrointestinal disease, and malignancy. This expanded etiological spectrum underscores the need for a broader clinical perspective. This review included studies indexed in EMBASE, PubMed, Scopus, Web of Science, and Google Scholar databases. Whether the tools were magnetic resonance imaging, diffusion tensor imaging, positron emission tomography, autopsy, or memory testing, the narrative stayed consistent: the mammillary bodies, thalamus, hippocampus, and cerebellum take the greatest hit. Imaging reveals symmetrical atrophy, micro-hemorrhages, and fraying white-matter tracts deeper in alcohol-related Wernicke-Korsakoff syndrome, while non-alcoholic cases show extracortical or cerebellar lesions. Clinically, presentations range from acute confusion and gait instability to subtle executive lapses, with severe anterograde amnesia marking the chronic stage. The best scanner cannot outmatch a timely intravenous dose of thiamine. Hours separate reversible Wernicke’s encephalopathy from lifelong Korsakoff syndrome. Building automatic thiamine checks into nutritional, surgical, oncological, and obstetric pathways and giving supplementation the moment deficiency is suspected can keep memories, independence, and quality of life intact.

## Introduction and background

The clinical and historical understanding of thiamine deficiency-related disorders has evolved significantly since their initial descriptions. Wernicke’s encephalopathy, first characterized by Carl Wernicke in 1881, was identified as a distinctive neuropsychiatric syndrome marked by the classic triad of ophthalmoplegia, ataxia, and mental confusion [1,2]. Interestingly, early case reports described the occurrence of Wernicke’s encephalopathy in both alcoholic and non-alcoholic individuals, thereby suggesting a broader clinical spectrum than initially assumed [3]. These results underscored the vulnerability of some brain regions to metabolic disturbances. Concurrently, Sergei Korsakoff reported a chronic amnestic syndrome, initially designated “alcoholic paralysis,” in individuals with long-standing alcoholic misuse, thereby advancing the definition of a syndrome that would later bear his name [4]. The convergence of these independent lines of investigation ultimately resulted in the identification of Wernicke-Korsakoff syndrome as one disease process with two discrete stages: an acute stage was linked to Wernicke’s encephalopathy, and a chronic stage was associated with Korsakoff syndrome [5]. While the traditional association of thiamine deficiency with chronic alcoholism is historically important, the range of conditions associated with this nutritional deficiency, including hyperemesis gravidarum, gastrointestinal diseases, and post-bariatric surgery syndromes, has expanded to include a variety of etiologies. This underscores the importance of recognizing thiamine deficiency as a potential complication in diverse patient populations [6]. Therefore, this review aims to synthesize current evidence on the neuroanatomical and cognitive sequelae of thiamine deficiency, with a focus on neuroimaging findings and their implications for diagnosis and treatment. Special emphasis is placed on the etiological diversity of Wernicke-Korsakoff syndrome and the importance of considering non-alcoholic causes in the differential diagnosis of acute encephalopathy and memory disorders [4].

## Review

In this narrative review, the search results from 1984 to 2025 in PubMed, EMBASE, Scopus, Web of Science, and Google Scholar were reviewed to track how thiamine deficiency alters the brain structures in Wernicke-Korsakoff syndrome. The articles were screened for data on structural, functional, and neuropathological changes. The findings were tabulated to follow the pattern of neuroanatomical changes, highlighting the differences between alcohol- and non-alcohol-related damage. The goal is simple yet urgent: to turn this evidence into earlier thiamine checks, faster treatment, and, ultimately, spared memories for patients who might otherwise slip into irreversible amnesia.

Thiamine (vitamin B1) is a cofactor that assists in the propagation of action potential along the axon during neurotransmission [7]. Vitamin B1 is absorbed in the small intestine. Thiamine has limited body stores, a short half-life, and is renally excreted, making regular intake essential. Deficiency arises when intake is inadequate or absorption/excretion is impaired. Patients present with neurologic, cardiac, and gastrointestinal symptoms, and, if prolonged or untreated, progress to Wernicke-Korsakoff syndrome [8]. Wernicke-Korsakoff syndrome is a serious neuropathologic syndrome induced by thiamine deficiency, most commonly associated with chronic alcohol abuse [9]. This syndrome encompasses two distinct clinical stages: Wernicke’s encephalopathy, an acute neurological emergency that may be reversible with timely intervention, and Korsakoff syndrome, a chronic and frequently irreversible amnestic disorder [10]. Although traditionally linked to alcoholism, Wernicke-Korsakoff syndrome has also been increasingly recognized in non-alcoholic patients, arising in the context of nutritional deficiencies due to bariatric surgery, hyperemesis gravidarum, gastrointestinal malignancies, and eating disorders [11,12]. Clinically, Wernicke’s encephalopathy is characterized by the classic triad of ophthalmoplegia, ataxia, and altered mental status or dementia; however, this triad is observed in fewer than one-third of the cases, contributing to significant rates of underdiagnosis [12]. When untreated, Wernicke’s encephalopathy progresses to Korsakoff syndrome, characterized by severe memory impairments of both anterograde and retrograde amnesia, confabulation, as well as confusion or other cognitive and psychomotor symptoms that lead to considerable disruption in daily cognitive functioning [13].

Neuroimaging has played a pivotal role in advancing the understanding of the structural brain alterations associated with thiamine deficiency. Magnetic resonance imaging (MRI) often reveals symmetric signal hyperintensities on fluid-attenuated inversion recovery (FLAIR) sequences in the thalami, mammillary bodies, periaqueductal gray matter, and tectal plate [14,15]. Additionally, modern neuroimaging techniques, including positron emission tomography (PET), computed tomography (CT), and MRI, have facilitated the differentiation between the acute and chronic stages of the syndrome [16]. MRI, particularly T2-weighted imaging/FLAIR and diffusion-weighted imaging (DWI), remains the optimal technique for detecting acute lesions, and volumetric T1-weighted imaging for chronic atrophy [17]. While CT scans are less sensitive than MRI in early detection, they may still serve a role in resource-limited settings due to greater availability and rapid acquisition times [18,19].

In alcoholic Wernicke-Korsakoff syndrome, widespread white matter degeneration, including damage to the corpus callosum and cerebellar peduncles, is commonly observed, whereas non-alcoholic presentations may involve atypical cortical and cerebellar lesions [14,20]. Neuropathological investigations of the disorder have consistently reported neuron loss, gliosis, and microhemorrhages in the affected gray matter regions, with particular involvement of the thalamus and mammillary bodies [3,21].

Sex-based differences in the neuropathological presentation of Wernicke-Korsakoff syndrome have also been identified [19]. In comparison, summarized by Hommer (2003), alcohol-related neuroanatomical changes were mixed in sexes: both men and women show global brain atrophy. Some studies have reported brain shrinkage and a smaller-sized corpus callosum in alcoholic women than in men or female controls; however, other studies found no shrinkage in alcoholic women, while men did. Further, the size of the hippocampus is reduced bilaterally in alcoholic women, while only the right-sided hippocampus is reduced in alcoholic men. Methodological factors such as age control, intracranial volume, analysis type, and drinking history may explain these discrepancies. Overall, women are more vulnerable, but the evidence is inconclusive [22]. Non-alcoholic Wernicke-Korsakoff syndrome tends to affect more women and young patients. Even when excluding cases related to hyperemesis gravidarum, there is still a slight female predominance with higher survival and recovery rates [19,23]. Males always exhibit alcohol-related Wernicke-Korsakoff syndrome changes, while females, particularly those with non-alcoholic Wernicke-Korsakoff syndrome, more often present with different atypical involvement [20,24-26]. These differences underscore the role of both biological and etiological factors in modulating disease progression and outcomes.

Despite its clinical severity and potential for reversibility, Wernicke-Korsakoff syndrome often remains underrecognized, especially in non-alcoholic and atypical presentations [27]. Delayed diagnosis may result in irreversible neurological damage and long-term cognitive impairment. Therefore, heightened clinical vigilance and prompt thiamine administration remain critical for preventing the progression from Wernicke’s encephalopathy to Wernicke-Korsakoff Syndrome [28,29].

Korsakoff syndrome: neuroanatomical changes

Korsakoff syndrome is characterized by a range of profound neuroanatomical alterations, largely attributed to thiamine deficiency, which results in irreversible cognitive impairment. Among the most consistently reported structural changes are those affecting the mammillary bodies, which are considered key structures within the posterior hypothalamus integral to memory circuits [30,31]. Histological and neuroimaging studies consistently demonstrate significant bilateral atrophy, with reductions in volume in 60-80% observed in 96-100% of patients [21,31]. These changes are frequently accompanied by gliosis and edema [9] and are strongly implicated in the hallmark episodic memory deficits of Korsakoff syndrome [21].

Although the chronic form of Korsakoff syndrome is characterized by atrophic changes, the acute form of Wernicke’s encephalopathy is characterized by the presence of bilateral symmetric edema in the mammillary bodies, thalamus, periaqueductal gray matter, and colliculi [31-34]. In Korsakoff syndrome, these regions often progress to atrophic degeneration, especially the thalamus, where both the anterior and mediodorsal nuclei are severely affected [21]. Quantitative studies have shown that a neuron loss in the mediodorsal thalamus appears to increase from 48% in Wernicke’s encephalopathy to 64% in Korsakoff syndrome [24], directly correlating with the severity of mnemonic and cognitive dysfunctions [35]. Further neuroanatomical compromise is evident in the hippocampus, where volume loss and white matter atrophy reflect widespread network disruption [36,37]. Cortical structures, including the frontal lobes, also exhibit significant atrophy, particularly in Korsakoff syndrome cases not associated with alcoholism [31,38]. This frontal lobe degeneration underpins the executive dysfunction and planning deficits commonly reported in these patients. Notably, neuronal density in the dorsolateral prefrontal cortex may be reduced by up to 20% in alcoholic individuals [21]. Comparison studies have highlighted that patients with Korsakoff syndrome experience more severe cognitive and anatomical impairments than those with chronic alcoholism alone [39]. White matter hyperintensities, a frequent radiological finding in Korsakoff syndrome, signify diffuse myelin disruption and impaired neural connectivity [13,19]. Research by Pitel et al. (2012) and Segobin et al. (2015) further reported more extensive white matter alterations in Korsakoff syndrome compared to those with chronic alcoholism, suggesting more advanced cerebral impairment [38,40].

The cerebellum, especially the vermis, is also impaired in Korsakoff syndrome, associated with ataxia and executive dysfunction [33,40]. Alcoholic individuals with Wernicke’s encephalopathy also exhibit a loss of Purkinje cells, which are crucial for coordinating motor function [20,41]. Thiamine deficiency not only causes structural disruption of the brain but also a neurochemical imbalance. Thiamine is involved in several metabolic pathways, including the biosynthesis of the important neurotransmitter acetylcholine, which is essential for memory and learning processes [26,42]. The resulting reduction in cholinergic transmission to the hippocampus and frontal cortex exacerbates the cognitive symptoms associated with Korsakoff syndrome [43].

Alcohol-related brain changes

The structural changes in the brain associated with chronic alcohol consumption differ in many aspects from those induced by thiamine deficiency [44]. Neuroimaging studies have identified significant alterations, including degeneration of the cerebellum. This heightened sensitivity of the cerebellum to alcohol’s detrimental effects contributes to motor dysfunction and impairments in cognitive processing, given the cerebellum’s crucial roles in coordinating movement and supporting various cognitive operations [26,34,45]. Unlike Korsakoff syndrome, patients with chronic alcohol use disorder commonly show greater brain atrophy, as neuronal damage can be more widespread in alcoholics, affecting many more brain areas [46]. Studies have shown that the negative effects on cognitive function resulting from alcohol-induced brain damage are not limited to memory problems alone but extend to a range of cognitive abilities, including, but not limited to, attention span, executive functions, and the capacity for impulse control [47,48]. The use of functional neuroimaging tools in studies of chronic alcohol users has demonstrated that the brain shows unusual activation during cognitive tasks, thus providing evidence for a compensatory role of brain activity in response to alcohol-induced neuronal damage [38,44,49].

Chronic alcoholism differently involves the frontal lobes, parietal lobes, and cerebellum [34]. The impaired cerebello-cortical networks may thus lead to neurocognitive impairments [26]. Additionally, the extent of cerebellar atrophy is proportionate to the severity of cognitive and motor impairments in alcohol-related syndromes. While the frontal lobe is affected in both Korsakoff syndrome and chronic alcoholism, the specific patterns and severity of degeneration may differ [33]. Individuals with alcohol use disorder can exhibit cognitive deficits similar to those seen in Korsakoff syndrome [50].

White matter integrity is also significantly compromised by chronic alcohol misuse. Diffusion tensor imaging studies have identified disrupted connectivity between the frontal lobe and other cortical and subcortical structures, which likely underpins many of the observed cognitive deficits [51]. These disruptions impair neural signal conduction and contribute to inefficient information processing, highlighting the importance of network-level alterations in alcohol-related brain damage [49]. Moreover, the impaired function of the mesolimbic dopaminergic system, which is involved in reward-related behaviors as well, can be a risk of severe alcoholism [20]. Chronic alcohol consumption exerts neurotoxic effects through various mechanisms, including oxidative stress and inflammatory processes, leading to neuronal damage. The combination of alcohol-related neurotoxicity and thiamine deficiency may contribute to increased brain damage in patients with both of these disease processes.

Non-alcoholic Wernicke-Korsakoff syndrome

Although Wernicke-Korsakoff syndrome is frequently associated with chronic alcoholism, a wide range of non-alcohol-related conditions can also precipitate thiamine deficiency and result in Wernicke-Korsakoff syndrome. Recent epidemiological analyses have demonstrated a disproportionately higher incidence of non-alcoholic Wernicke-Korsakoff syndrome in females, with a male-to-female ratio of approximately 1:1.84 [19]. The various non-alcoholic causes of Wernicke-Korsakoff syndrome can stem from multiple medical illnesses and nutritional deficiencies. Gastrointestinal disorders, such as cancer, obstruction, and pancreatitis, can contribute to thiamine deficiency, resulting in Wernicke-Korsakoff syndrome [1,4,11]. Notably, cancers such as cholangiocellular carcinoma have been implicated, particularly when associated with gastric outlet obstruction or the need for prolonged parenteral nutrition without adequate thiamine supplementation [52]. Similarly, hyperemesis gravidarum represents a significant risk factor due to persistent vomiting and consequent nutrient depletion [11,53,54]. Chronic malabsorption and starvation, whether due to psychiatric illnesses such as anorexia nervosa and schizophrenia spectrum disorders or due to gastrointestinal diseases, such as Crohn’s disease or ulcerative colitis, can also lead to thiamine deficiency and the onset of Wernicke-Korsakoff syndrome [1,55]. Bariatric surgery has been increasingly recognized as another critical risk factor. The altered gastrointestinal anatomy post-surgery may impair nutrient absorption, making patients vulnerable to Wernicke-Korsakoff syndrome if not carefully monitored and supplemented [1].

Moreover, cases of thalamic amnesia, which clinically resemble Wernicke-Korsakoff syndrome, have been reported following infarction or damage localized to the left thalamus [21]. Various types of cancer, particularly those affecting the lymphoid system, along with conditions such as intravenous feeding or hyperalimentation without adequate thiamine replacement, are associated with the development of Wernicke-Korsakoff syndrome [1,4,19]. In addition to schizophrenia spectrum disorders and anorexia nervosa [1,55], severe depression, especially when accompanied by psychosis and self-neglect, along with significant weight loss, can induce Wernicke-Korsakoff syndrome without necessarily being related to alcohol abuse [56,57]. Other comorbidities, such as dialysis, human immunodeficiency virus/acquired immunodeficiency syndrome, and different underlying problems, have also been associated with non-alcoholic Wernicke-Korsakoff syndrome [1,19,56]. These diverse etiologies highlight the necessity for heightened clinical vigilance in identifying thiamine deficiency and Wernicke-Korsakoff syndrome in populations not traditionally associated with alcohol use. Clinicians must remain alert to the risks in patients with pre-existing diseases that predispose them to malnutrition, malabsorption, or increased metabolic demand, to prevent irreversible neurological damage. Another uncommon finding of Wernicke’s encephalopathy/Wernicke-Korsakoff syndrome can occasionally mimic the stroke MRI changes that show extensive bilateral frontal cortical involvement along with the classic midline lesions [58]. In cases of bilateral thalamic lesions accompanied by meningeal enhancement, it is essential to consider potential alternative or overlapping diagnoses such as dengue meningoencephalitis rather than Wernicke’s encephalopathy [59].

Wernicke-Korsakoff syndrome in infants, children, and adolescents

In comparison with adults, the most common cause of thiamine deficiency in infants and young children is malnutrition. Brain injuries often have prominent basal ganglia and frontal lobe involvement in addition to the classic thalami, mammillary bodies, periaqueductal gray, and tectal plate [60,61]. In adolescents, the primary cause is eating disorders, prolonged vomiting, and total parenteral nutrition; the main neuroanatomical changes can be atypical or initially unremarkable. Hence, treatment should be started immediately, even without confirmation of the diagnosis [62,63]. By contrast, adults show clear alteration of the medial thalami, mammillary bodies, periaqueductal region, and tectal plate, with white matter change in chronic stages. However, the adult pattern can occur in older children. Wernicke-Korsakoff syndrome cannot be excluded in normal radiology. This underscores the need to deliberately check other atypical areas such as the basal ganglia along with immediate thiamine administration [14,64].

Table 1 and Table 2 distil the evidence base uncovered in our 1984-2024 literature sweep. Table 1 synthesizes neuroimaging and neuropathology findings, linking each vulnerable brain region in acute Wernicke’s encephalopathy and chronic Korsakoff syndrome to its structural signature and functional deficit. Table 2 represents the documented non-alcoholic triggers of thiamine-deficiency encephalopathy, mapping etiology to the neural targets, bedside presentation, and expected outcome with prompt replacement therapy. Together, they translate dozens of heterogeneous studies into a concise, at-a-glance reference for clinicians and researchers.

**Table 1:** Neuroanatomical changes in Wernicke’s encephalopathy and Korsakoff syndrome. The table illustrates the brain regions that are most affected in acute Wernicke’s encephalopathy when compared to chronic Korsakoff syndrome. It includes the imaging or histological techniques used to detect these changes (such as MRI, DTI, FDG-PET, or postmortem analysis) and indicates whether the findings represent a reduction in volume or fiber integrity. WE = Wernicke’s encephalopathy; KS = Korsakoff syndrome; MRI = magnetic resonance imaging; DTI = diffusion tensor imaging; FDG-PET = fluorodeoxyglucose-positron emission tomography

Studies	Brain region	Tissue	WE	KS	Structural change (direction/extent)	Modality	Functional impact	Level of evidence
Sullivan and Pfefferbaum (2008) [31]; Jung et al. (2012) [33]; Kril and Harper (2012) [21]	Mammillary bodies	Gray	✓	✓	Atrophy, gliosis; 60–80% volume loss	MRI, postmortem	Severe anterograde amnesia	Cross‑sectional MRI + histology
Kopelman (2014) [37]; Arts et al. [24] (2017); Nardone et al. (2013) [9]	Anterior and mediodorsal thalamus	Gray	✗	✓	Lesions, atrophy, ↓ metabolism	MRI, FDG‑PET	Executive dysfunction, confabulation	Cross‑sectional MRI and PET
Oscar‑Berman et al. (2004) [65]; Zahr et al. (2011) [20]	Orbitofrontal cortex	Gray	✗	✓	Cortical thinning, white matter loss	MRI, PET	Disinhibition, poor decision‑making	Cross‑sectional MRI + PET
Zahr et al. (2011) [20]; Beresford et al. (2006) [66]	Hippocampus (CA2/3)	Gray	✗	✓	Regional atrophy (≈15–25%)	MRI	Episodic memory deficits	Cross‑sectional MRI
McCormick et al. (2011) [67]; Oudman et al. (2015) [68]	Corpus callosum (splenium)	White	✗	✓	Demyelination, ↓ FA	DTI	Slow interhemispheric transfer	DTI case‑control
Nardone et al. (2013) [9]; Day et al. (2013) [69]	Pons/Midbrain tegmentum	Mixed	✓	✗	Lesions in the ascending RAS	MRI	Arousal dysfunction	Case series MRI
Nardone et al. (2013) [9]	Cingulate gyrus	Gray	✗	✓	Posterior atrophy, hypometabolism	PET	Apathy, attentional control	Cross‑sectional PET
Nardone et al. (2013) [9]; Fama et al. (2012) [70]; Di Lorenzo et al. (2022) [71]	Cerebral cortex (frontal/temporal)	Mixed	✗	✓	Diffuse thinning, white matter degeneration	MRI	Global cognitive decline	MRI review
Nardone et al. (2013) [9]; Victor et al. (1989) [72]	Cerebellar vermis	Gray	✓	✓	Purkinje cell loss, folial atrophy	MRI, Histology	Gait ataxia, exec. deficits	Histology + MRI
Oudman et al. (2015) [68]	Fornix	White	✗	✓	Axonal degeneration, volume loss	MRI, DTI	Memory consolidation failure	DTI cohort
Zahr et al. (2011) [20]; Logan et al. (2016) [34]	Periaqueductal gray	Gray	✓	✗	Edema, micro‑hemorrhages	MRI (T2)	Autonomic dysregulation	Case series MRI
Logan et al. (2016) [34]	Tectal plate	Gray	✓	✗	Bilateral hyper‑intensity	MRI	Ocular movement deficits	Case report MRI
Kril and Harper (2012) [21]	Hypothalamus	Gray	✓	✓	Mammillary nucleus lesions	MRI	Endocrine/autonomic dysfunction	MRI review
Nardone et al. (2013) [9]	Basal ganglia (caudate/putamen)	Gray	✗	✓	Volume loss, motor circuit disruption	MRI, PET	Motor incoordination	Cross‑sectional MRI + PET
Beresford et al. (2006) [66]; Pitel et al. (2009) [38]	Medial temporal lobes	Gray	✗	✓	Entorhinal cortex atrophy	MRI	Episodic memory decline	Cross‑sectional MRI

**Table 2:** Non‑alcoholic causes of Wernicke-Korsakoff syndrome. This table highlights non-alcoholic causes of the Wernicke-Korsakoff spectrum to underscore that thiamine deficiency can occur outside of chronic alcohol use. Conditions such as bariatric surgery, prolonged hyperemesis, malignancy, inflammatory bowel disease, and refeeding syndrome can contribute to this deficiency. The table outlines the pathophysiological mechanisms, including malnutrition, malabsorption, and increased demand, along with the associated clinical presentations and potential outcomes for each of these conditions. WE = Wernicke’s encephalopathy; KS = Korsakoff syndrome; CNS = central nervous system; ICU = intensive care unit; UC = ulcerative colitis; HIV/AIDS = human immunodeficiency virus/acquired immunodeficiency syndrome

Etiology (non‑alcoholic cause)	CNS structures commonly involved	Core clinical presentation	Typical outcomes (with timely thiamine)	Key reference(s)
Bariatric surgery (gastric bypass, sleeve)	Mammillary bodies, thalami, cerebellar vermis	Confusion, ophthalmoplegia, ataxia; anterograde memory loss emerging weeks postoperatively	Excellent neurological recovery if IV thiamine within 24 hours; persistent amnesia if delayed	Oudman et al. (2021) [1]
Hyperemesis gravidarum	Thalami, mammillary bodies, periaqueductal gray	Intractable vomiting → confusion, nystagmus, gait unsteadiness	Marked improvement; occasional mild residual cognitive deficits	Accetta et al. (2002) [54]; Souza et al. (2023) [53]
Total parenteral nutrition without thiamine	Mammillary bodies, thalami	Acute encephalopathy with ocular palsy in ICU/oncology setting	Rapid reversal when recognized; >20% mortality if diagnosis missed	Scalzo et al. (2015) [19]
Gastrointestinal malignancy/Obstruction	Mammillary bodies, thalami	Delirium, diplopia, truncal ataxia in cachectic patients	Partial neurological recovery; overall prognosis tied to primary cancer	Koca et al. (2022) [52]
Inflammatory bowel disease (Crohn’s/UC)	Mammillary bodies, thalami	Confusion, broad‑based gait, horizontal gaze palsy	Good outcome with supplementation; relapse risk with active disease	Oudman et al. (2021) [1]
Anorexia nervosa/Restrictive eating disorders	Cerebellar vermis, frontal cortex, mammillary bodies	Cognitive clouding, oculomotor signs, postural instability	Variable, persistent executive‑memory deficits are common	Langlois et al. (2014) [55]; Oudman et al. (2021) [1]
Bulimia nervosa (repeated vomiting)	Mammillary bodies, thalami	Acute Wernicke triad superimposed on electrolyte imbalance	Generally good with thiamine and nutritional restoration	Isenberg‑Grzeda et al. (2012) [29]
Refeeding syndrome after prolonged starvation	Mammillary bodies, thalami, brainstem	Rapid cognitive decline during high‑carbohydrate refeeding	Preventable with prophylactic thiamine; otherwise, risk of chronic KS	Chandrakumar et al. (2018) [13]
Chronic dialysis (hemodialysis/peritoneal)	Mammillary bodies, thalami, cerebellum	Fluctuating confusion, nystagmus peri‑dialysis	Reversible with dose‑adjusted supplementation	Scalzo et al. (2015) [19]
HIV/AIDS‑associated malnutrition	Mammillary bodies, cerebral cortex	Subacute confusion, ocular signs, gait ataxia	The outcome depends on immune recovery; treatable neurologically	Scalzo et al. (2015) [19]
Pancreatitis‑induced prolonged fasting/Starvation	Mammillary bodies, thalami	Delirium, ocular motor deficits, limb ataxia	Good neurological recovery when treated early	Wijnia (2022) [11]; Eva et al. (2023) [4]
Psychiatric illness with self‑neglect (major depression, schizophrenia)	Mammillary bodies, thalami	Apathy, confusion, profound amnesia	Cognitive sequelae frequent; psychiatric follow‑up essential	Saraiva et al. (2021) [56]; Cocksedge and Flynn (2014) [57]
Chronic loop‑diuretic therapy/Heart failure	Mammillary bodies, thalami	Confusion, ataxia after brisk diuresis	Excellent recovery with thiamine and diuretic dose review	Isenberg‑Grzeda et al. (2012) [29]
Chemotherapy‑induced malabsorption	Mammillary bodies, thalami	Acute ocular palsy, confusion during cytotoxic cycles	Reversible; consider prophylactic thiamine in high‑risk regimens	Hudak et al. (2014) [18]
Celiac disease/Chronic malabsorption	Mammillary bodies, thalami	Progressive gait ataxia, diplopia, confusion	Responds to thiamine and gluten‑free diet	Bae et al. (2016) [44]

## Conclusions

Although alcohol abuse remains the primary cause of Wernicke-Korsakoff syndrome, an increasing number of non-alcoholic cases can now be related to gastrointestinal disease, malabsorption, and other medical disorders. The application of new methodologies, such as DTI and high-resolution MRI, has broadened our knowledge about the microstructural changes that occur in the brain in alcoholic and non-alcoholic conditions. Nonetheless, there are significant limitations in acknowledging the extent of cognitive impairments and their long-term consequences. Future research should aim to enhance early identification, delineate diagnostic criteria, and refine personalized rehabilitation strategies. These are vital efforts to reduce the impact of cognitive impairment on affected individuals.
